# Effects of 12-Week Sorghum Consumption on Visceral Fat Area and Metabolic Parameters in Japanese Adults: An Exploratory Single-Arm Trial

**DOI:** 10.3390/nu18121884

**Published:** 2026-06-11

**Authors:** Hitomi Miyazaki, Masumi Nagae, Akiko Isa, Yuko Takano, Hiroshi Uchida, Kuniyoshi Shimizu

**Affiliations:** 1 Division of Sustainable Bioresources Science, Department of Agro-Environmental Sciences, Faculty of Agriculture, Graduate School of Kyushu University, 744 Motooka, Nishi-ku, Fukuoka 819-0395, Japan; 2 Best Amenity Co., Ltd., 32-3 Tagawa, Mizuma-machi, Kurume 830-0102, Japan; 3 Kyushu University Institute of Asian and Oceanic Studies, Kyushu University, 744 Motooka, Nishi-ku, Fukuoka 819-0395, Japan

**Keywords:** sorghum, visceral fat area, abdominal obesity, whole grains, metabolic parameters, Japanese adults, bioelectrical impedance analysis

## Abstract

**Background:** Visceral fat accumulation is strongly associated with metabolic disorders, particularly in Japanese adults who accumulate visceral fat even at lower body mass index levels. Sorghum is a whole grain rich in resistant starch and polyphenols, which may influence visceral fat area (VFA). This exploratory study aimed to investigate the effects of 12-week sorghum consumption on VFA and metabolic parameters in Japanese adults with visceral fat accumulation. **Methods:** This single-arm intervention trial included adults aged 20–60 years with VFA ≥ 100 cm^2^ and no ongoing medical treatment. Participants consumed cooked sorghum (80 g/day, dry weight) for 12 weeks. Anthropometric variables, VFA, blood pressure, and blood biomarkers were assessed before and after the intervention. Dietary intake was evaluated using a three-day food record. Pre- and post-intervention values were compared using paired *t*-tests. **Results:** Nine participants completed the study. VFA significantly decreased after 12 weeks of sorghum consumption (*p* = 0.02). Alanine aminotransferase (ALT) levels showed a non-significant reduction, while other metabolic and hepatic biomarkers remained stable. No adverse changes were observed in dietary intake or physical activity. Eight of nine participants exhibited reductions in VFA. **Conclusions:** Daily sorghum consumption may contribute to reductions in VFA and improvements in liver-related biomarkers in Japanese adults with visceral fat accumulation. These findings provide preliminary evidence that partially replacing major carbohydrate sources with sorghum may support visceral fat management. Further confirmation in randomized controlled trials is warranted.

## 1. Introduction

Excessive visceral fat accumulation is strongly associated with lifestyle-related risk factors, such as impaired glucose tolerance, dyslipidemia, and hypertension. These conditions contribute to the development of atherosclerosis and cardiovascular events. Visceral adipose tissue secretes various bioactive proteins collectively known as adipokines, and dysregulation of adipokine secretion in obesity promotes the onset and progression of lifestyle-related diseases.

Analyses of body fat distribution using abdominal computed tomography (CT) have revealed that visceral obesity is associated with a higher risk of metabolic abnormalities and atherogenic cardiovascular disease than subcutaneous obesity [1,2]. Visceral fat area (VFA) is positively correlated with the number of cardiovascular risk factors, whereas subcutaneous fat area shows no such association [1]. When VFA exceeds 100 cm^2^, the average number of risk factors surpasses one in both men and women; thus, VFA ≥ 100 cm^2^ is widely used as a diagnostic criterion for visceral obesity [2].

East Asians, including Japanese individuals, accumulate higher levels of visceral fat than Western populations, even at comparable body mass index (BMI) levels [3,4]. This ethnic difference has been proposed as a potential explanation for the higher susceptibility of Japanese adults to obesity-related diseases compared with Western populations [3,4].

Sorghum is one of the major cereal grains worldwide and has been used as food and feed since its origin in Africa. In recent years, sorghum has gained attention as a sustainable, health-promoting, gluten-free grain, characterized by low water and fertilizer requirements and a high content of dietary fiber and antioxidant compounds [5]. In Japan, sorghum has long been cultivated as “takakibi,” and its value as a functional grain is being re-evaluated, with scientific evidence supporting expectations for market expansion.

Sorghum is a whole grain rich in dietary fiber, polyphenols, and tannins. Previous studies in animals and humans have shown that sorghum intake contributes to weight reduction and improves glycemic control [6,7,8,9,10]. Sorghum consumption lowers postprandial glucose and insulin levels in healthy adults [9]. Continuous sorghum intake improves blood lipid profiles and contributes to weight reduction [6,8]. However, most clinical studies have been conducted in Africa, the United States, and Australia; evidence in Japanese populations remains limited. In particular, no clinical trials have examined the effects of sorghum consumption on VFA in Japanese adults. To the best of our knowledge, this is the first study to demonstrate that 12-week sorghum consumption significantly reduced VFA in Japanese adults with visceral fat accumulation.

Therefore, this exploratory study aimed to investigate the effects of 12-week sorghum consumption on VFA and related metabolic parameters in Japanese adults with visceral fat accumulation.

## 2. Materials and Methods

### 2.1. Study Design

This study was a 12-week, single-arm intervention trial designed to evaluate changes in VFA following daily sorghum consumption. The study was conducted at Shiba Palace Clinic (Fukuoka, Japan). The trial was registered with the UMIN Clinical Trials Registry (UMIN000052564, 23 October 2023).

### 2.2. Participants

Participants were recruited through Asmark Inc., which invited volunteers from its registered panel. Individuals who expressed interest were informed about the study’s purpose. Approximately 20 candidates who provided preliminary consent were initially listed.

Eligibility was assessed using predefined inclusion and exclusion criteria. Inclusion criteria were: Japanese men and women aged 20–60 years with a waist circumference ≥ 85 cm (men) or ≥90 cm (women). These waist circumference criteria were established for initial screening to identify individuals with potential visceral fat accumulation. Exclusion criteria included: ongoing treatment for lifestyle-related diseases, ongoing treatment for medical conditions, history of severe illness, habitual use of supplements, food or drug allergies, participation in other clinical trials within the past month, heavy alcohol consumption (>60 g/day), smoking, and any condition judged inappropriate by the study physician.

After screening, 10 individuals with visceral fat area (VFA) ≥ 100 cm^2^ who met all the criteria were selected as eligible participants. Written informed consent was obtained from the participants by the principal investigator prior to enrollment. One participant withdrew for personal reasons; ultimately nine participants were finalized for the analysis.

Baseline lifestyle characteristics—including smoking status, alcohol intake, and habitual dietary patterns—were assessed using a structured questionnaire, and no changes in these lifestyle factors were reported during the intervention period.

### 2.3. Intervention

Participants consumed one pack of cooked sorghum daily (80 g dry weight) for 12 weeks. The test food was provided in retort pouches by Best Amenity Co., Ltd. (Kurume, Fukuoka, Japan). The nutritional composition of the sorghum product is presented in Table 1.

Participants were instructed to maintain their typical diet and physical activity levels during the study. Adherence to daily sorghum consumption was monitored using participant intake logs and through confirmation at each study visit. Compliance was predefined as the consumption of at least 80% of the total allotted doses during the 12-week intervention. All nine participants met this criterion, and the mean adherence rate was 98.5%.

### 2.4. Outcome Measures

#### 2.4.1. VFA

VFA was measured using a belt-type bioelectrical impedance analysis (BIA) device manufactured by Panasonic Corp. (Osaka, Japan). This device estimates abdominal VFA from electrical impedance measurements obtained around the waist. All measurements were performed in a standing position after a brief rest, following the manufacturer’s instructions. This belt-type abdominal BIA method has been validated against CT-derived VFA in Japanese adults and has been shown to provide significantly correlated estimates [11,12].

#### 2.4.2. Anthropometric and Clinical Parameters

Body weight, body fat percentage, and BMI were measured using a calibrated body composition analyzer. Blood pressure was measured in a seated position after several minutes of rest using an automated sphygmomanometer. Blood biomarkers—including lipid profiles, glucose metabolism markers, and liver enzymes—were assessed at baseline and at week 12. Renal function was examined using serum creatinine levels, which were measured as part of the standard biochemical analyses. Notably, estimated glomerular filtration rate (eGFR) was not calculated.

Blood samples were collected in the university laboratory after an overnight fast (from 10:00 p.m. on the previous day until completion of the examination on the following morning). All biochemical analyses were outsourced to LSI Medience Corporation (Tokyo, Japan), a certified clinical testing laboratory.

### 2.5. Dietary Assessment

Dietary intake was assessed using a three-day food record before and after the intervention. Records were analyzed using the CAND^®^ dietary assessment system (ORTHOMEDICO Inc., Tokyo, Japan), which has been validated for use in Japanese populations.

### 2.6. Statistical Analysis

Statistical analyses were performed using EZR version 1.63, a graphical interface for R (version 4.3.1) [13]. Paired *t*-tests (two-tailed) were used to compare pre- and post-intervention values for VFA, anthropometric variables, blood biomarkers, and nutrient intake. This test was selected as corresponding measurements were taken from the same participants at two time points, resulting in dependent data. The normality of the difference scores was confirmed through visual inspection of histograms and Q–Q plots, justifying the assumptions required for the paired *t*-test. Given the exploratory nature of the study and the very small sample size, effect size estimates were not reported because they would introduce instability and be potentially misleading. A significance level of *p* < 0.05 was applied. Missing data were not imputed.

### 2.7. Ethical Approval

The study was conducted in accordance with the Declaration of Helsinki and approved by the Ethics Committee of Shiba Palace Clinic (protocol code: 153335_rn-36032). All participants provided written informed consent prior to enrollment.

## 3. Results

One participant withdrew for personal reasons, leaving nine participants for the final analysis. Participant characteristics are shown in Table 2.

The mean age of the participants was 43.3 ± 5.87 years, and the mean baseline VFA was 164.6 ± 34.5 cm^2^.

After 12 weeks of sorghum consumption, VFA significantly decreased compared with baseline values (*p* = 0.02). No significant changes were observed in body weight or BMI (Table 3).

**Figure 1:**
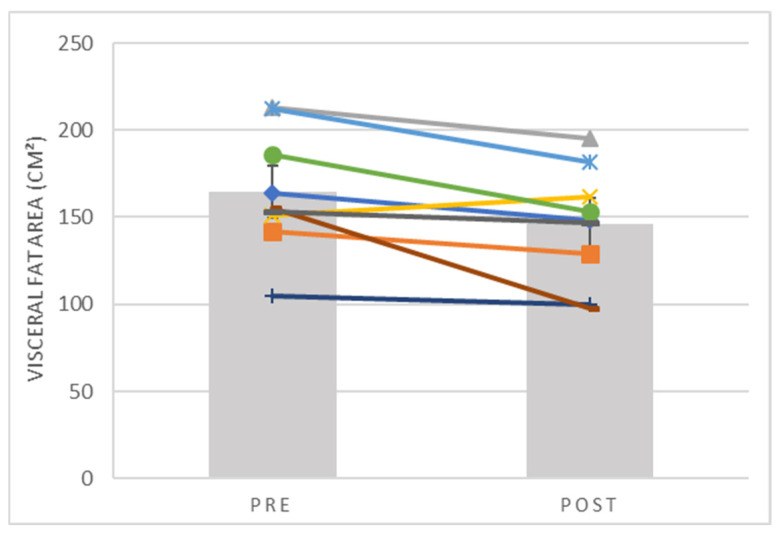
Individual changes in visceral fat area (cm^2^) before and after 12 weeks of sorghum consumption. Each colored line represents one participant. Gray bars indicate group mean values with 95% confidence intervals (mean change: −18.6 cm^2^; 95% CI: −33.3 to −3.9). Visceral fat area decreased significantly after the intervention (*p* = 0.02, paired *t*-test). Abbreviation: cm^2^, square centimeters.

Individual changes in VFA are presented in Figure 1.

Eight of the nine participants showed a reduction in VFA after the intervention, whereas one participant showed a slight increase. The overall decrease in VFA was statistically significant (*p* = 0.02), supporting the primary outcome of this study.

Baseline triglyceride levels were elevated (174.7 ± 75.9 mg/dL) and decreased by approximately 15% after the intervention, although the change was not statistically significant. Alanine aminotransferase (ALT) levels also decreased by approximately 20% from baseline, but this change was not statistically significant. No adverse changes were observed in other metabolic or hepatic biomarkers during the intervention period (Table 4).

No adverse metabolic changes were observed during the intervention. All metabolic and hepatic biomarkers remained stable, and none showed clinically remarkable deterioration. ALT levels exhibited a decreasing trend, while other parameters, including lipid and glucose metabolism markers, did not worsen (Table 4). Renal function also remained stable throughout the intervention. Serum creatinine levels did not show any clinically significant changes, indicating that the intervention did not adversely affect the renal function.

Dietary intake and physical activity levels remained stable throughout the study.

## 4. Discussion

This exploratory single-arm trial demonstrated that 12 weeks of sorghum consumption were associated with a significant reduction in VFA in Japanese adults with visceral fat accumulation. Although body weight and BMI did not change, the decreased VFA suggests that sorghum intake may influence abdominal fat distribution independently of overall weight change. In addition, ALT levels decreased marginally (*p* = 0.08), enabling potential improvements in hepatic or lipid metabolism.

The statistically significant decrease in VFA observed in this study indicates a reduction in visceral fat rather than mere random variation. Visceral fat accumulation contributes to metabolic abnormalities through increased free fatty acid flux and adipokine dysregulation. Therefore, the reduction in VFA may reflect improvements in metabolic function, an interpretation further supported by the observed trend toward lower ALT levels.

Beyond its statistical significance, the reduction in visceral fat has important clinical implications. Visceral fat is strongly associated with insulin resistance, dyslipidemia, hypertension, and chronic inflammation, all major drivers of cardiometabolic risk. Thus, even a modest decrease in VFA may improve metabolic health and potentially lower the risk of cardiovascular and cerebrovascular diseases. These findings align with previous evidence suggesting that whole-grain consumption improves metabolic health and reduces cardiometabolic risk.

Although abdominal BIA is practical and non-invasive, it provides an indirect estimate of visceral fat area and is subject to measurement variability compared with computed tomography (CT), which is considered the gold standard. Therefore, the changes in VFA observed in this study should be interpreted with these methodological limitations in mind.

No adverse events related to the intervention were reported during the study period, and no clinically relevant changes were observed in biochemical safety parameters, including liver and renal function markers. These findings indicate that the 12-week sorghum intervention was well tolerated and demonstrates a favorable safety profile.

Because participants were recruited based on having a VFA ≥ 100 cm^2^, the cohort included individuals with both mild metabolic abnormalities and metabolically healthy individuals. This heterogeneity may have attenuated detectable changes in metabolic biomarkers despite the significant reduction in VFA.

To contextualize the present findings, we compared our findings with previous whole-grain and sorghum-based intervention studies, particularly randomized controlled trials conducted in Asian populations [14]. Several RCTs have demonstrated that whole-grain intake can reduce visceral adiposity and improve metabolic parameters in Japanese and other Asian cohorts. Notably, a randomized double-blind trial in Japanese adults showed that replacing refined wheat bread with whole-grain wheat bread for 12 weeks significantly reduced VFA (measured by CT), supporting the relevance of the VFA reduction observed in the present study.

Whole grains have been widely reported to reduce cardiometabolic risk, improve gut microbiota composition, and lower all-cause mortality [15,16]. Dietary patterns rich in whole grains, such as the Mediterranean and DASH diets, have been shown to reduce visceral adiposity [17], which is consistent with the present findings. Furthermore, a randomized, double-blind trial in Japanese adults demonstrated that replacing refined wheat bread with whole-grain wheat bread for 12 weeks significantly reduced VFA measured by CT [18], supporting the potential of whole-grain interventions to reduce visceral fat in Japanese populations.

Several mechanisms may explain the observed reduction in VFA. Sorghum is rich in resistant starch, polyphenols, and condensed tannins, which may influence adiposity through multiple pathways. Resistant starch has been shown to reduce visceral fat, increase adiponectin, decrease leptin, and improve gut microbiota composition in animal models [10]. Sorghum polyphenols may also inhibit adipocyte differentiation by downregulating PPARγ and C/EBPα [7]. In addition, fermentation of resistant starch increases short-chain fatty acid (SCFA) production, which may activate GPR41/43, enhance fatty acid oxidation, and increase GLP-1 and PYY secretion [19]. Improvements in gut microbiota composition have also been reported in human trials using sorghum flour [15]. Although mechanistic biomarkers were not measured in this study, these pathways may collectively contribute to reductions in visceral fat.

The decrease in ALT levels observed in this study may reflect improvements in hepatic lipid metabolism. Visceral fat accumulation contributes to hepatic dysfunction through increased delivery of free fatty acids and pro-inflammatory adipokines [2]. A clinical study in patients with non-alcoholic steatohepatitis reported that reductions in visceral fat were accompanied by increased adiponectin and decreased liver enzyme levels [20]. Although the ALT reduction in this study was not statistically significant, the observed trend is consistent with these findings. The reduction in triglyceride (TG) levels did not reach statistical significance, likely due to the small sample size and substantial inter-individual variability in TG levels. Baseline TG levels varied widely among participants, ranging from normal to elevated levels, which may have restricted our capacity to detect significant changes. In addition, TG levels are highly sensitive to acute dietary changes, which contributes to data variability and attenuation of statistical power.

Taken together, the present results suggest that daily sorghum consumption may reduce visceral fat through multiple mechanisms, including modulation of adipokines, inhibition of adipocyte differentiation, improvements in gut microbiota composition, and increased SCFA production. However, these mechanisms remain hypothetical and require confirmation in future mechanistic studies. It should be noted that the mechanistic explanations discussed—including potential effects on adipokines, gut microbiota, SCFA production, and GLP-1 signaling—remain hypothetical and are inferred from prior literature, as these specific biomarkers were not evaluated in the current study. Therefore, these interpretations are exploratory and require further validation in future mechanistic investigations.

### Limitations

This study has several limitations. First, the small sample size and the absence of a control group limit the ability to attribute the observed changes solely to sorghum consumption. Given the small sample size, statistical power was limited, increasing the risk of both type I and type II errors. Therefore, these findings should be interpreted as exploratory. Effect sizes were omitted because the small sample size would yield highly unstable estimates that could be easily misinterpreted. Second, the 12-week intervention period was relatively short, and the long-term sustainability of the effects remains unknown. Third, dietary intake and physical activity were assessed using self-reported questionnaires, which may be subject to recall and reporting bias. Fourth, because the participants were Japanese adults aged 20–60 years with visceral fat accumulation, the generalizability of the findings to other age groups, ethnicities, or individuals without visceral obesity is limited. Finally, mechanistic biomarkers such as adipokines, inflammatory markers, and gut microbiota composition were not measured, preventing direct evaluation of the pathways through which sorghum may influence visceral fat. In addition, because this study employed a single-arm exploratory design without a control group, definite causal inferences cannot be drawn. Unmeasured lifestyle or behavioral changes during the intervention period may also have contributed to the observed reduction in visceral fat area. Consequently, larger randomized controlled trials are warranted to confirm the causal effects of sorghum intake.

## 5. Conclusions

This exploratory single-arm trial suggests that 12 weeks of sorghum consumption may reduce VFA and improve liver-related biomarkers in Japanese adults with visceral fat accumulation. However, the absence of a control group limits causal interpretation. Daily sorghum intake may represent a simple and sustainable dietary approach for individuals at risk of metabolic disorders. The present findings provide preliminary information that may support the design of future randomized controlled trials, including considerations for intake levels, intervention duration, and outcome selection. Larger, well-controlled studies are needed to confirm these findings and clarify the underlying mechanisms. These findings may help inform dietary recommendations for individuals with visceral obesity.

## Figures and Tables

**Table 1:** Nutritional composition of the test food (80 g dry weight of sorghum).

Nutrient	Amount per 80 g (Dry Weight)
Energy (kcal)	275
Protein (g)	8.2
Fat (g)	3.8
Carbohydrate (g)	56.9
Dietary fiber (g)	7.8

Values are expressed per 80 g of dry weight.

**Table 2:** Baseline characteristics of the participants.

Characteristic	Value (Mean ± SD)
N (M/W)	9 (6/3)
Age	43.3 ± 5.87
Body weight (kg)	80.6 ± 7.66
BMI (kg/m^2^)	27.5 ± 1.54
Body fat (%)	31.4 ± 7.98
Visceral fat area (cm^2^)	164.6 ± 34.5
Waist circumference (cm)	100.4 ± 5.73
SBP (mmHg)	123.8 ± 14.1
DBP (mmHg)	91.3 ± 8.16

Values are presented as mean ± standard deviation (SD). Abbreviations: BMI, body mass index; DBP, diastolic blood pressure; M/W, men/women; SBP, systolic blood pressure.

**Table 3:** Changes in anthropometric parameters before and after the intervention.

Variable	Pre (Mean ± SD)	Post (Mean ± SD)	*p*-Value
Body weight (kg)	80.6 ± 7.66	81.1 ± 8.16	0.301
BMI (kg/m^2^)	27.5 ± 1.54	27.6 ± 1.51	0.359
Body fat (%)	31.4 ± 7.98	31.6 ± 7.73	0.813
Visceral fat area (cm^2^)	164.6 ± 34.5	146.0 ± 33.0	0.020
Waist circumference (cm)	100.4 ± 5.73	98.7 ± 5.17	0.129
SBP (mmHg)	123.8 ± 14.1	124.6 ± 15.4	0.812
DBP (mmHg)	91.3 ± 8.16	89.6 ± 13.4	0.721

Values are presented as mean ± standard deviation (SD). *p*-values were calculated using paired *t*-tests.

**Table 4:** Changes in blood biomarkers before and after the intervention.

	Pre (Mean ± SD)	Post (Mean ± SD)	*p*-Value
TC (mg/dL)	205.6 ± 30.7	198.8 ± 35.5	0.301
HDL-C (mg/dL)	48.3 ± 11.7	46.3 ± 13.6	0.523
LDL-C (mg/dL)	128.3 ± 31.3	128.2 ± 31.5	1.000
TG (mg/dL)	174.7 ± 75.9	148.4 ± 77.8	0.203
Non-HDL-C (mg/dL)	157.2 ± 29.1	152.4 ± 31.6	0.441
FBG (mg/dL)	90.1 ± 7.36	94.9 ± 8.94	0.122
Insulin (µU/mL)	7.11 ± 1.71	7.26 ± 3.82	0.734
HbA1c (%)	5.43 ± 0.24	5.44 ± 0.26	0.750
HOMA-IR	1.59 ± 0.42	1.75 ± 1.05	0.833
AST (IU/L)	25.9 ± 8.72	24.7 ± 6.73	0.722
ALT (IU/L)	48.2 ± 35.3	38.8 ± 22.3	0.080
γ-GTP (IU/L)	57.2 ± 46.2	54.0 ± 47.2	0.137
UA (mg/dL)	5.84 ± 1.20	5.87 ± 1.36	0.910
UN (mg/dL)	12.6 ± 3.33	12.5 ± 3.11	0.859
Cr (mg/dL)	0.828 ± 0.09	0.831 ± 0.09	0.953
TP (g/dL)	7.04 ± 0.11	7.26 ± 0.08	0.094
ALB (g/dL)	4.53 ± 0.05	4.48 ± 0.07	0.457

Values are presented as mean ± standard deviation (SD). *p*-values were calculated using paired *t*-tests. Abbreviations: TC, total cholesterol; HDL-C, high-density lipoprotein cholesterol; LDL-C, low-density lipoprotein cholesterol; TG, triglyceride; Non-HDL-C, non-high-density lipoprotein cholesterol; FBG, fasting blood glucose; HbA1c, hemoglobin A1c; HOMA-IR, homeostasis model assessment of insulin resistance; AST, aspartate aminotransferase; ALT, alanine aminotransferase; γ-GTP, gamma-glutamyl transpeptidase; UA, uric acid; UN, urea nitrogen; Cr, creatinine; TP, total protein; ALB, albumin.

## Data Availability

The data supporting the findings of this study are available from the corresponding author upon reasonable request. The data are not publicly available due to privacy and ethical restrictions.

## Abbreviations

The following abbreviations are used in this manuscript:

ALT	Alanine aminotransferase
AST	Aspartate aminotransferase
ALB	Albumin
BIA	Bioelectrical impedance analysis
BMI	Body mass index
BP	Blood pressure
cm^2^	Square centimeters
Cr	Creatinine
CT	Computed tomography
DBP	Diastolic blood pressure
FBG	Fasting blood glucose
GLP-1	Glucagon-like peptide-1
HbA1c	Hemoglobin A1c
HDL-C	High-density lipoprotein cholesterol
HOMA-IR	Homeostasis model assessment of insulin resistance
LDL-C	Low-density lipoprotein cholesterol
Non-HDL-C	Non-high-density lipoprotein cholesterol
PYY	Peptide YY
SBP	Systolic blood pressure
SCFAs	Short-chain fatty acids
SD	Standard deviation
TC	Total cholesterol
TGs	Triglycerides
UA	Uric acid
UN	Urea nitrogen
VFA	Visceral fat area
γ-GTP	Gamma-glutamyl transpeptidase
